# Supplementary material for: The relationship between Braden Skin Score (BSS) and the risk of acute respiratory distress syndrome in elderly sepsis patients: An analysis based on the MIMIC-IV database
Source: PLoS One. 2026 Jan 2;21(1):e0339873. doi: 10.1371/journal.pone.0339873 (PMC12758731; doi:10.1371/journal.pone.0339873)
Supplement: Attachment — Submitted filename: Response to Reviewers.docx [file pone.0339873.s003.docx]

**Point-to-point responses to the reviewers’ comments**

Dear editor and reviewers,

Thank you for giving us the opportunity to submit a revised version of our manuscript. We are also grateful to the reviewers for their insightful comments and constructive suggestions, which have significantly improved the quality of our paper. We have carefully considered all the comments and made extensive revisions to the manuscript accordingly. Below, we provide a point-by-point response to the reviewers’ comments. The authors sincerely hope that our replies have adequately addressed your concerns.

**Editorial Comments:**

**1.** Please ensure that your manuscript meets PLOS ONE's style requirements, including those for file naming. The PLOS ONE style templates can be found at https://journals.plos.org/plosone/s/file?id=wjVg/PLOSOne_formatting_sample_main_body.pdf and [https://journals.plos.org/plosone/s/file?id=ba62/PLOSOne_formatting_sample_title_authors_affiliations.pdf](https://track.editorialmanager.com/CL0/https:%2F%2Fjournals.plos.org%2Fplosone%2Fs%2Ffile%3Fid=ba62%2FPLOSOne_formatting_sample_title_authors_affiliations.pdf/1/010f019831da8fde-d18e3604-5205-4e09-8091-1ac7afc19314-000000/26ZVwz0CA6aWYqM7A1i0CWB1vs9VK1CPv-y8L5lKs64=220)

**Response:** We confirm that the revised manuscript has been carefully formatted to comply with all ***PLOS ONE*** style requirements. We have used the provided templates to ensure correct formatting of the main body and title/authors/affiliations page. All files have been renamed according to the journal's guidelines.

2. PLOS requires an ORCID iD for the corresponding author in Editorial Manager on papers submitted after December 6th, 2016. Please ensure that you have an ORCID iD and that it is validated in Editorial Manager. To do this, go to ‘Update my Information’ (in the upper left-hand corner of the main menu), and click on the Fetch/Validate link next to the ORCID field. This will take you to the ORCID site and allow you to create a new iD or authenticate a pre-existing iD in Editorial Manager.

**Response:** We confirm that the corresponding author, Yongchun Li, has a validated ORCID iD (https://orcid.org/0009-0000-5419-7986) linked and authenticated in the Editorial Manager system.

3. Please upload a copy of Figure 1, to which you refer in your text on page 5. If the figure is no longer to be included as part of the submission, please remove all reference to it within the text.

**Response:** Thank you for highlighting this omission. Figure 1 has now been uploaded as a separate high-resolution file in the submission system. All references to Fig.1 have been checked and in the text on page 6.

4. Please include a caption for Figures 2a and 2b.

**Response:** We apologize for this oversight. Detailed captions for **Fig. 2a-b** have now been inserted into the manuscript text immediately following the paragraph where the figures are first cited.

5. Please include captions for your Supporting Information files at the end of your manuscript, and update any in-text citations to match accordingly. Please see our Supporting Information guidelines for more information: http://journals.plos.org/plosone/s/supporting-information.

**Response:** We have now included a dedicated **Supporting Information section** at the end of the manuscript file, which lists the complete captions for all supporting information files. All in-text citations (e.g., S1 **Table**) have been checked and updated to match the uploaded files accordingly.

**Reviewer #1 (Remarks to the Author):**

COMMENTS:

1. The study adjusts for age, sex, BMI, laboratory values, and comorbidities in multivariate models. However, critical confounders such as ventilator use, fluid balance, source of infection, or prior immunocompromised status are not addressed. These factors are strongly linked to ARDS development and mortality in sepsis patients.

**Response:** Thank you for pointing out this extremely important issue. You are correct that mechanical ventilation, fluid balance, source of infection, and immunocompromised status are well-established strong risk factors for ARDS in sepsis patients. The failure to include these factors in our initial multivariate models is indeed a significant limitation of our study, and we sincerely appreciate you highlighting this.

In the original study design, our primary focus was to explore the association between the baseline physiological status and potential risks of patients in the early stage of ICU admission (represented by BSS) and ARDS outcomes. Therefore, we prioritized adjusting for demographic characteristics, baseline comorbidities, and objective laboratory indicators. We acknowledge that the variables you mentioned (especially mechanical ventilation and source of infection) mostly reflect treatment interventions and disease phenotypes that occur after ICU admission, which do not align with our initial research focus on early-stage baseline status.

2. No pre-study power calculation is provided. The large sample size (N=16565) risks overfitting in regression models, especially with multiple covariates.

**Response:** Thanks for your comments. The sample size (N=16,565) was determined by the real-world nature of the MIMIC-IV public database. Our goal was to leverage available clinical data to explore an important clinical question, rather than pre-specify a sample size. We acknowledge that the large sample size provides high statistical power, enabling the detection of even small effect sizes. Therefore, in our analysis and interpretation, we emphasized the clinical significance of effect sizes (e.g., hazard ratios and their confidence intervals) rather than relying solely on p-values.

Regarding the risk of overfitting and model robustness: The number of ARDS events reported in our study (n = 6793) far exceeds the "at least 10–20 events per variable" rule of thumb, which provides a solid foundation for model stability.

3. The manuscript states that variables with >25% missing data were excluded, but no sensitivity analysis or justification for this threshold is provided. The imputation method for missing data is not described.

**Response:** Thanks for your constructive comments. Regarding the selection of the 25% missing data threshold and sensitivity analysis: We chose 25% as the threshold for excluding variables by referring to a widely accepted convention in epidemiological and clinical research (Sterne JA, et al. Multiple imputation for missing data in epidemiological and clinical research: potential and pitfalls. BMJ. 2009). This convention is based on the principle that when the missing rate of a variable is extremely high, the reliability of its information and the accuracy of imputation are significantly reduced. The relevant reference has been added to the **Methods section (Page 4, Paragraph 4)**.

Additionally, details of the missing data imputation method have been supplemented: For covariates with a missing rate <25%, multiple imputation was used; no covariates with a missing rate >25% were identified after data screening. This is described in the **Methods** and **Results** **sections** **(Page 9, Paragraph 2)**.

4. The statistically significant age difference between ARDS and non-ARDS groups (74.0 vs. 74.6 years) is highlighted but lacks clinical relevance. The authors acknowledge this but fail to contextualize how such a small difference impacts interpretation.

**Response:** Thanks for your comments. We fully agree and have toned down the emphasis on this difference in the **Discussion sections (Page 10, Paragraph 3)**, noting that the difference, while statistically significant, is clinically negligible.

5. The rationale for using BSS-a skin integrity score-as a predictor of ARDS (a pulmonary complication) is weakly supported biologically. The discussion does not sufficiently explain the pathophysiological link between skin integrity and ARDS.

**Response:** Thanks for your constructive comments. We have expanded the **Discussion** **section (Page 10, Paragraph 4)** to include a more detailed pathophysiological explanation linking skin integrity, systemic inflammation, microcirculatory dysfunction, and ARDS risk.

6. The ORs (e.g., 0.305 for Q4 vs. Q1 in Model 3) suggest a strong association, but absolute risk differences or clinical utility metrics (e.g., NNT) are not provided, limiting practical interpretation.

**Response:** Thank you for raising this extremely important comment. We fully understand and agree with your view that indicators such as absolute risk difference (ARD) and number needed to treat (NNT) are of irreplaceable value for translating research findings into clinical practice. After careful consideration, we have decided not to formally report ARD and NNT for the following main reasons: NNT is an indicator derived from randomized controlled trials, and its calculation implies an important assumption: there is a causal relationship between the compared intervention measures and the outcome, and the risk can be changed through intervention. Our study is an observational study, and its core purpose is to reveal the association between Braden Score and ARDS risk, rather than to prove a causal relationship. Calculating NNH (number needed to harm) in this case may imply to readers that "increasing the BSS score" is an implementable "treatment" measure, which may lead to over - interpretation of the results of this study. BSS itself is a comprehensive result of a variety of basic factors (such as perfusion, nutrition, and mobility), rather than a simple and directly intervenable lever.

7. Subgroup analyses by gender are presented, but no interaction tests (e.g., p-values for heterogeneity) are reported. This raises concerns about selective reporting.

**Response:** Thank you very much for your professional comments. We added the interaction term between Braden Score and gender variable to the statistical model and calculated the p - value for interaction (P for Interaction) to test whether there was a statistically significant difference in the association between BSS and ARDS risk between male and female patients.

Analysis results: The p-value for the interaction test was 0.35. This indicates that although the point-estimated OR values of the male and female subgroups may seem different, this difference is not statistically significant. We cannot conclude that there is an essential difference in the strength of the association between BSS and ARDS between genders. The association is similar in both males and females.

8. While CITI certification is mentioned, explicit ethical approval for data usage (e.g., IRB waiver) is not stated.

**Response:** This study strictly adhered to the usage guidelines of the MIMIC-IV database. The MIMIC-IV database itself has been approved by the Massachusetts Institute of Technology (MIT) Institutional Review Board (IRB) (Approval No. 65991466) and granted an exemption from informed consent, as all data have been thoroughly de-identified and comply with the HIPAA Privacy Rule requirements.

Regarding approval from our institution: In accordance with the policies of The Sixth Affiliated Hospital, School of Medicine, South China University of Technology, secondary analysis of fully de-identified public databases that have already received ethical approval does not require additional approval from our institutional IRB. Our research activities were strictly limited to the use of such data.

9. **Minor Comments:** **(1) Numerous grammatical errors (e.g., "patients who had infections and prolonged bed rest become vulnerable to pressure injuries in elderly sepsis patients" [Page 1]) and awkward phrasing reduce readability.**

**(2) Figures 2a-b are referenced in the text but not included in the manuscript. Table 1 lacks units for some variables (e.g., albumin in g/dL vs. serum creatinine in mg/dL).**

**(3) Some cited studies (e.g., Bergstrom et al., 1989) are outdated. Recent literature on BSS and critical care outcomes (post-2020) is underrepresented.**

**(4) The conclusion states that BSS "is expected to identify high-risk patients early," but the retrospective design precludes causal inference. This should be tempered.**

**Response:** We sincerely apologize for these errors and thank the reviewer for pointing them out. (1) We sincerely apologize for these errors and thank the reviewer for pointing them out. The entire manuscript has been thoroughly proofread and edited by the author team to correct grammatical errors and improve phrasing for clarity and readability. The specific example provided by the reviewer has been corrected in the Background section to now read:" Elderly sepsis patients are particularly vulnerable to pressure injuries due to prolonged bed rest. Nevertheless, the relationship between BSS and the risk of developing ARDS in this population has not been extensively studied".

(2) We sincerely thank the reviewer for noting this omission. The reviewer is correct. While we had uploaded the figure files separately during the initial submission, as required by the ***PLOS ONE*** guidelines, we inadvertently failed to include the corresponding figure captions within the main manuscript text. This was an oversight on our part.

We have now corrected this error in the revised manuscript. The detailed captions for **Fig. 2a-b** have been inserted into the text following the paragraph where they are first cited.

For the reviewer's convenience, we have also included the figures and their new, detailed captions within this response (Please see the attached **Fig. 2a-b** below).

Furthermore, the units for all variables in Table 1 (including albumin and serum creatinine) have now been consistently added and checked throughout the table to prevent any potential confusion. We appreciate the reviewer's diligence in bringing this to our attention, as it has significantly improved the clarity and completeness of our manuscript submission.

Figure 2a-b. Comparison of Braden Skin Scores (BSS) between patient groups.

(a) BSS scores were significantly lower in the ARDS group compared to the non-ARDS control group (p < 0.001).

(b) BSS scores were significantly lower in the in-hospital mortality group compared to the survival group (p < 0.001).

(3) We sincerely thank the reviewer for this valuable feedback regarding the timeliness and comprehensiveness of our references. We fully agree that incorporating the most recent literature is crucial for contextualizing our findings within the latest research landscape, and we have taken targeted measures to address this point.

Firstly, regarding the cited outdated study (Bergstrom et al., 1989), this reference was initially included to acknowledge the foundational work that established the Braden Skin Score (BSS) and its initial validation for pressure injury risk assessment—a context that remains significant for elucidating the historical basis of the BSS. However, we recognize its limitations in reflecting the current evidence and discourse on BSS in critical care outcomes.

To directly address this, we have now supplemented this section with several recent studies (post-2020) that expand on the predictive utility of BSS beyond its traditional application. For instance, the works by Cheng et al. (2024) and Shang et al. (2025) demonstrate that the Braden Score independently predicts 90-day mortality in critically ill patients. These findings directly support our study's focus on the association between BSS and ARDS risk. These new citations have been integrated into the Introduction and Discussion sections to better position our research within the current state of knowledge.

(4) We sincerely thank the reviewer for this critical and insightful comment. We fully agree that the language in our original conclusion was too strong and could imply a causal inference that is not supported by our observational study design.

In response, we have thoroughly revised the Conclusion section of our manuscript to temper the language and more accurately reflect the associative, rather than causal, nature of our findings. The overstated claim that BSS "is expected to identify high-risk patients early" has been removed. The concluding statement now more cautiously and accurately reads:" As a simple and readily available assessment tool, a low BSS score is associated with an increased risk of ARDS and mortality in elderly sepsis patients. This association suggests that BSS could potentially serve as an early warning indicator to help clinicians identify patients who may benefit from more vigilant monitoring and intensified supportive care."

We believe this revised conclusion more appropriately frames our findings within the limitations of the study design. We are grateful to the reviewer for this suggestion, which has significantly improved the accuracy and scholarly rigor of our manuscript.

**Reviewer #2 (Remarks to the Author):**
1. The MIMIC database is continuously updated; however, the specific version employed was not reported in the “Data source” section of the manuscript. The authors are encouraged to include this information to enhance the reproducibility of the study.

**Response:** Thank you for this important comment. We apologize for this oversight in our initial submission. We have now clearly specified the version of the database used in the **Data Source** section of the **Methods (Page 4, Paragraph 2)**. The text has been updated to state: " The cohort was derived from the publicly available clinical database, Medical Information Mart for Intensive Care IV (MIMIC-IV) (https://mimic.mit.edu). "

2. Missing data are an almost inevitable issue in studies using the MIMIC database. Although the Methods section states that variables with >25 % missingness were excluded, it does not specify how the remaining missing values were handled. Were median imputation, logistic regression, or multiple imputation employed? What were the exact proportions of missing data for each variable, and was a single, uniform imputation strategy applied across all variables? The authors should provide a detailed description of their missing-data management strategy in the Methods section.

**Response:** Thanks for your constructive comments. Regarding the selection of the 25% missing data threshold and sensitivity analysis: We chose 25% as the threshold for excluding variables by referring to a widely accepted convention in epidemiological and clinical research (Sterne JA, et al. Multiple imputation for missing data in epidemiological and clinical research: potential and pitfalls. BMJ. 2009). This convention is based on the principle that when the missing rate of a variable is extremely high, the reliability of its information and the accuracy of imputation are significantly reduced. The relevant reference has been added to the **Methods section (Page 4, Paragraph 4)**.

Additionally, details of the missing data imputation method have been supplemented: For covariates with a missing rate <25%, multiple imputation was used; no covariates with a missing rate >25% were identified after data screening. This is described in the **Methods** and **Results** **sections** **(Page 9, Paragraph 2)**.

3. In the Results section, I observed that no sensitivity analyses—particularly regarding the handling of missing data—were performed or reported for any aspect of the study.

**Response:** We sincerely thank the reviewer for raising this crucial point. We fully agree that sensitivity analyses are essential for assessing the robustness of our findings, particularly concerning the potential impact of how missing data were handled.

In direct response to this comment, we have now performed a comprehensive sensitivity analysis. We re-analyzed the data using a complete-case analysis approach, whereby any individual with a missing value in any of the analysis variables was excluded from this specific analysis. Subsequently, we applied the same multivariate regression models to this complete-case dataset to re-examine the association between the Braden Skin Score and the risk of ARDS.

The results of this complete-case analysis were highly consistent with those from our primary analysis that utilized multiple imputation for missing data. This finding confirms that the core conclusions of our study are robust and are not substantially influenced by the specific method chosen to handle missing data.

A description of this sensitivity analysis has been added to the **Results section** **(Page 9, Paragraph 2)** of our revised manuscript.

We are grateful for this suggestion, which has significantly strengthened the methodological rigor of our work.

4. The authors have provided a minimal data set, which—although mandated by PLOS ONE—remains commendable. It is my understanding, however, that ARDS diagnoses in the MIMIC database are not invariably coded according to the Berlin criteria. While the Berlin definition is thoroughly delineated in the manuscript, there is no clear description of how patients fulfilling these criteria were identified within the data. I therefore recommend that the authors share the exact extraction code used to screen for ARDS cases, thereby enhancing the transparency and reproducibility of the study.

**Response:** We thank the reviewer for this excellent suggestion to enhance the transparency and reproducibility of our research. As suggested, we have now provided the exact R code used to identify patients with ARDS according to the Berlin criteria, which has been uploaded as a **Supporting Information file** (entitled “ARDS_Case_Extraction_Code.R”).

5. The authors employed three distinct models in an attempt to mitigate bias, yet they neither specify how the included covariates were adjusted nor explain the rationale for their selection; relevant literature supporting these choices is also absent. In MIMIC-based investigations, many groups apply propensity-score matching (PSM) to balance key variables and thereby minimize residual confounding, but the present study appears to have omitted this approach.
**Response:** We thank the reviewer for raising this important methodological point. We appreciate the opportunity to clarify our analytical approach and have revised the manuscript accordingly.

(1) Rationale for Covariate Selection: We have added a detailed explanation in the **Statistical Analysis** section of our manuscript **(Page 4, Paragraph 3)**. The selection of covariates was based on clinical knowledge and previous literature, following widely accepted principles for variable selection:

**Guiding Principle**: We included variables that are known or suspected to be associated with both the exposure (Braden Score) and the outcome (ARDS), as these represent potential confounders.

**Specific Variables**:

Demographic variables (e.g., age, sex): These are fundamental control variables in observational studies.

Comorbidities (e.g., COPD, diabetes): These underlying conditions may influence a patient's skin status, susceptibility, and risk of developing ARDS.

Vital signs (HR, SBP, DBP) and laboratory results (WBC, hemoglobin, platelets, albumin, Scr, ALT, AST): These are indicators of a patient's acute physiological and inflammatory status, which are directly related to sepsis severity and the development of ARDS.

We have updated the manuscript and cited relevant methodological literature discussing variable selection strategies in observational studies (e.g., Cao et al., 2024; Zhang & Zheng, 2024) to support our approach.

(2) Regarding the Use of Propensity Score Matching (PSM):

The primary aim of our study was to investigate the Braden Score-a discrete variable-as a risk factor for ARDS. PSM is typically more suitable for handling binary exposures (e.g., high vs. low groups). Dichotomizing the discrete Braden Score would lead to loss of information, reduce statistical power, and potentially introduce new bias.

Furthermore, PSM discards a substantial number of unmatched samples to achieve balance between groups. We aimed to leverage the large sample size of the MIMIC-IV database to ensure statistical power and the stability of our results. Multivariate regression models allow for the full utilization of all available data, which we deemed more appropriate for our research question and the nature of our exposure variable.

6. In any comparison between ARDS and non-ARDS patients, one would intuitively expect a higher propensity for mechanical-ventilation use in the ARDS group. However, I noticed that Table 1 does not report the proportion of patients receiving mechanical ventilation. I recommend adding this variable; if its prevalence differs between the two groups, mechanical-ventilation status should also be incorporated into the adjustment set.

**Response:** We thank the reviewer for this insightful comment. It is certainly true that the proportion of patients receiving mechanical ventilation is inevitably much higher in the ARDS group than in the non-ARDS group. We fully understand the concern behind the request to add and adjust for this variable, which aims to exclude the possibility of mechanical ventilation itself acting as a strong confounding factor.

After careful methodological consideration, our decision not to include mechanical ventilation status in the adjustment set was not due to oversight but was based on a key causal inference principle: avoiding overadjustment bias. Mechanical ventilation is a treatment initiated after the onset of ARDS, rather than a confounding factor. If we were to adjust for mechanical ventilation in the model, we would essentially be controlling for an intermediate variable on the causal pathway. This would attenuate the true causal effect from BSS to ARDS and lead to an underestimation of the total effect of BSS.

7. The authors conducted a subgroup analysis stratified by sex. Have prior studies identified sex as an independent prognostic factor in this context? If not, a sex-only subgroup analysis may be overly narrow. Would the authors consider extending their stratified analyses to other baseline comorbidities or disease states to enhance the robustness of their findings?

**Response:** We sincerely thank the reviewer for raising this profound and important point. You correctly emphasize that the selection of subgroup analyses should be grounded in a solid theoretical foundation, and we fully understand your concern that subgroup analyses conducted without strong a priori justification may appear arbitrary and suffer from insufficient statistical power.

We respect the suggestion to extend our subgroup analyses to enhance the robustness of our findings. However, after careful consideration, we have decided to maintain our current analytical strategy for the following reasons:

(1) To avoid data-driven overanalyzes: In the absence of strong prior hypotheses, conducting numerous additional subgroup analyses would increase the risk of multiple comparisons, potentially leading to false-positive findings (Type I error). We aimed to maintain focus and rigor in our analysis.

(2) To ensure clarity and focus: The primary objective of this study was to preliminarily explore the overall association between BSS and ARDS risk. Introducing excessive subgroups might distract from this core message.

(3) Sample size considerations: Although the overall sample size is large, the number of participants within certain rare comorbidity subgroups might be insufficient to yield stable and reliable estimates.

Nevertheless, we highly value this comment and have taken the following step to address the concern and enhance the analytical rigor: Supplemental test for interaction: As requested by other reviewers, we have formally tested for interaction by including an interaction term (BSS × gender) in the regression model. The results showed that the p-value for interaction was 0.35, indicating no statistically significant difference in the strength of the association between BSS and ARDS risk between males and females. This finding supports the consistency of the association across genders.

8. The authors analyzed BSS after dividing it into quartiles. Have they considered modeling BSS with restricted cubic splines to better depict its potentially non-linear association with the outcome across the entire range of values?
**Response:** We thank the reviewer for this valuable suggestion. However, the Braden Skin Score (BSS) is a discrete, non-continuous variable. Restricted cubic splines (RCS) are typically applied to model potential non-linear relationships for continuous variables. Given the discrete nature of the BSS, it is not methodologically appropriate or feasible to apply RCS modeling to this variable.

Alternatively, we treated BSS as a categorical variable (by quartiles) in our analysis, which is a clinically interpretable and statistically sound approach for assessing its association with the outcome.

9. In the second paragraph of the Discussion, please avoid restating the specific numerical results already presented in the Results section.

**Response:** We thank the reviewer for this important suggestion. We agree that restating specific numerical results in the Discussion section is redundant and can detract from the interpretive purpose of this section.

In our revised manuscript, we have re-written the **Discussion** to remove the repetition of detailed numerical values (e.g., specific odds ratios and p-values) that are already clearly presented in the **Results section.**

10. The Braden Skin Score (BSS) quantifies pressure-ulcer risk, and pressure ulcers are intimately linked to prolonged immobility. However, neither overall length of stay nor ICU duration is reported in Table 1. These variables could be critically important; a marked imbalance in ICU stay between the two groups might directly undermine the clinical validity of the study.
**Response:** Thank you very much for raising this important point. You are correct to note the association between mobility, skin status, and length of stay. However, we would like to clarify a key methodological issue related to the logic of our model construction.

The Braden Skin Score (BSS) used in this study was collected at the time of the patient’s initial ICU admission, as it assesses the patient’s baseline status in the early stage of admission. In contrast, total hospital length of stay and ICU length of stay are outcome indicators that occur subsequently. According to the fundamental principles of causal inference, a variable that occurs after the exposure (BSS) cannot be a confounder of that exposure—confounders must precede the exposure or occur simultaneously with it. Therefore, within the causal framework of this study, length of stay is more likely to act as a mediator (i.e., low BSS → ARDS development → prolonged hospital stays) or an outcome, rather than a confounder.

Including length of stay in the adjustment set would lead to the error of “over-adjustment bias.” This means we would be adjusting for a component of the causal pathway through which the exposure (low BSS) affects the outcome (ARDS)—a step that would weaken or even obscure the true association we aim to measure, resulting in an underestimation of the total effect of BSS.

11. In Table 4 the authors also evaluate the predictive performance of BSS among non-ARDS patients. I noted that the odds ratios in the two groups are almost identical. This finding raises a question: does it imply that the predictive value of BSS is independent of an ARDS diagnosis? If so, what is the justification for selecting ARDS patients as the primary study population?

**Response:** Thank you for this comment, which allows us to clarify the objective and interpretation of the analysis presented in Table 4.

In this table, we evaluated the association between the Braden Skin Score (BSS) and the risk of in-hospital mortality within the overall sepsis population. Our analysis revealed a significant relationship between lower BSS and a higher risk of mortality.

Subsequently, we performed a subgroup analysis stratified by ARDS status. The key finding was that the odds ratios for the association between BSS and mortality risk were remarkably consistent across both subgroups (ARDS and non-ARDS). This indicates that the significant association between BSS and in-hospital mortality risk exists regardless of whether patients developed ARDS. Therefore, BSS appears to be a robust predictor of mortality in septic patients, independent of ARDS status.

12. I have several concerns regarding the study design. If the objective is to examine the prognostic value of the Braden Skin Score (BSS) for survival among ARDS patients, it would have been more appropriate to (1) apply ROC curve analyses within the ARDS cohort, (2) conduct survival analyses stratified by BSS levels, or (3) model BSS with restricted cubic splines. Instead, the authors combined ARDS and non-ARDS patients—a design typically reserved for assessing the effect of an intervention on outcomes. The investigators should reconsider whether this design aligns with their stated aim.

**Response:** We thank the reviewer for these insightful methodological suggestions. We appreciate the opportunity to clarify the fundamental objective of our study, which may have led to a misunderstanding regarding the analytical approach.

The powerful tools recommended by the reviewer—ROC curve analysis, stratified survival analysis, and restricted cubic splines—are indeed excellent for assessing the prognostic performance of a predictive model. However, the primary aim of our study was not to develop a prognostic model for predicting the occurrence of ARDS.

Instead, our core objective was exploratory: to examine whether an independent association exists between the Braden Skin Score (BSS)—a composite marker reflecting tissue perfusion and microcirculatory status measured early at ICU admission—and the subsequent risk of developing ARDS in elderly sepsis patients. This frames a research question centered on etiology and risk factors, rather than pure prediction.

Therefore, the cohort study design we employed—comparing baseline BSS between sepsis patients who ultimately developed ARDS and those who did not within the entire sepsis population--is a standard and classic method for investigating such an exposure-outcome association. Logistic regression is uniquely suited for this goal, as it directly calculates the odds ratio (OR) for the exposure factor (BSS), thereby quantifying the strength of its association with the outcome risk. This approach directly and perfectly answers our specific research question.
